# Analysis of the Machining Process of Inconel 718 Parts Manufactured by Laser Metal Deposition

**DOI:** 10.3390/ma12132159

**Published:** 2019-07-05

**Authors:** Txomin Ostra, Unai Alonso, Fernando Veiga, Mikel Ortiz, Pedro Ramiro, Amaia Alberdi

**Affiliations:** 1 TECNALIA, Parque Científico y Tecnológico de Gipuzkoa, 20009 Donostia-San Sebastián, Spain; 2 Department of Mechanical Engineering, University of the Basque Country (UPV/EHU), 48013 Bilbao, Spain

**Keywords:** additive manufacturing, hybrid manufacturing, laser metal deposition, directed energy deposition

## Abstract

Laser metal deposition (LMD) is an additive manufacturing process that allows the manufacturing of near-net-shape products. This could mean significant savings in terms of materials and costs in the manufacturing of high-performance components for the aeronautical industry. In this work, an analysis of how the LMD processing of alloy 718 affects the final machining has been carried out. For this purpose, a comparative study has been done by means of the monitoring of the end milling process of a part manufactured by LMD and a rough-milled part from forged material. Differences between process outputs such as chip morphology and cutting forces were studied. Material characteristics such as microstructure, hardness and mechanical properties were also analyzed.

## 1. Introduction

Laser metal deposition (LMD), also known as directed energy deposition (DED), laser cladding, or laser engineered net shaping (LENS), is an additive manufacturing (AM) process that uses a laser beam as an energy source. The laser beam creates a melt pool in a metallic substrate, where the material is added as powder or in a wire form. Afterwards, the added material is fused and solidified, creating a high-quality metallurgical union between the substrate and the added material. In this process, the material addition is usually made layer by layer and allows one to add material in areas where a very complex geometry is required, to repair a damaged area, or to generate a coating of a material different from the substrate. The final geometry of the created part is determined by the LMD head path.

One of the main advantages of LMD technology is that it can help to create near-net-shape products, e.g., by creating adapted pre-forms for forging by adding material to typical bar stock. This approach could mean significant savings in terms of materials and costs in the manufacturing of high-performance components for the aeronautical industry, which are commonly manufactured by forging to obtain the pre-form and then by machining a high quantity of material to obtain the desired final geometry. A widely used material for the manufacturing of this type of component is nickel-based superalloys, such as alloy 718, thanks to their exceptional mechanical resistance at high temperature and corrosive environments [1].

Due to the poor surface finish and low dimensional accuracy obtained during LMD, all these applications usually require a finishing step to obtain the geometrical and mechanical requirements [2]. For example, the average roughness value (Ra) resulting from LMD processes is often over 10 μm [2], and in mechanical systems, much smoother surfaces are needed. There can be found different approaches for the finishing, such as laser re-melting [2] or laser polishing [3], to improve the surface roughness significantly. These techniques can significantly improve the smoothness of the surface, but they are not valid for achieving the required dimensional accuracy (often under ISO IT10). Therefore, the most common approach is to finish the additively manufactured parts by an end-machining process. 

An industrial solution that is becoming very relevant is to combine both processes (LMD and machining) on the same platform, which maintains the advantages of AM techniques (form freedom, versatility, time to market, etc.) while also meeting the finishing requirements of machined parts [4,5]. The integration of LMD and machining in the same machine tool presents a series of drawbacks and limitations that are still not solved, and this aspect impacts directly on the industrialization of these systems [6]. 

The new paradigm of hybrid manufacturing makes it necessary to understand how the additive manufacturing process affects the machining operation. In this sense, the mechanical and chemical properties of the parts manufactured by additive manufacturing [7,8], and in particular by LMD [9], are very different from the forged ones. For example, the residual stresses may affect the end-machining and can lead to unexpected part distortions. Heigel et al. [7] observed this effect in an experimental study consisting of machining AISI 17-4 stainless steel cylinders manufactured by the direct metal laser sintering (DMLS) technique. It should also be considered that the residual stresses can affect the chip formation process. Lane et al. [8] observed curly chip formation during the machining of AISI 17-4 stainless steel disks also produced by DMLS. The effect of the LMD technology on chip geometry has not yet been studied for nickel-based superalloys. However, unfavorable residual stresses have been detected in, for example, Waspaloy [10], so similar results might be obtained. Oyelola et al. [9] also pointed out that other aspects related with the workpiece quality, such as surface roughness, could also be affected by the AM process. 

Inconel 718 is a nickel-based alloy that offers a high strength and toughness together with high temperature capability and environment degradation resistance. Yet, due to its high alloy content, it resists plastic deformation during machining and leads to rapid strain hardening [11]. Under these conditions, the machining process often causes the formation of residual stresses that lead to dimensional instability. Moreover, a short tool life, low material removal rates, and high machining costs are to be expected when processing such nickel-based alloys. In this regard, it has been observed that the wear of the cutting edge is mainly produced by abrasion, adhesion, and oxidation [12]. Other works have also reported several strategies for milling Inconel 718, related with the optimization of cutting parameters and tool geometry [13], cutting forces [14], or cooling systems [15]. 

Some works have also evaluated the machinability of alloy 718 manufactured by laser deposition. For example, Parimi et al. reported that the machining was also affected by the deposition strategy and the resulting microstructure [16]. In this case, the as-deposited microstructure of alloy 718 showed a columnar grain structure, growing epitaxially from the substrate, with the orientation of the grains. Furthermore, the morphology of the Laves phase also depends on the heat input and the cooling rate of the deposition process. The detrimental Nb-rich intermetallic Laves phase is formed in the interdendritic regions and embrittles the material [17]. Deposition processes with lower heat input and higher cooling rates produce lower fractions of the Laves phase, and consequently, better material properties [18,19]. Gonzalez et al. [20] performed a comparative analysis of powder-bed-based additive manufacturing (AM) technologies during the production of metallic components using Inconel 625 powder material. Despite the efforts of previous works, a comparison of the milling process of walls produced by LMD and forging has not been reported. 

The main objective of this work is to investigate the influence of the LMD process of Inconel 718 on the end-milling operation and to compare it to the machining of a forged workpiece. More specifically, a comparison of the mechanical properties and microstructure of both materials is done, and process outputs such as chip geometry and cutting forces are analyzed.

## 2. Materials and Methods

### 2.1. Experimental Setup for Hybrid Additive/Subtractive Manufacturing

**Figure 1:**
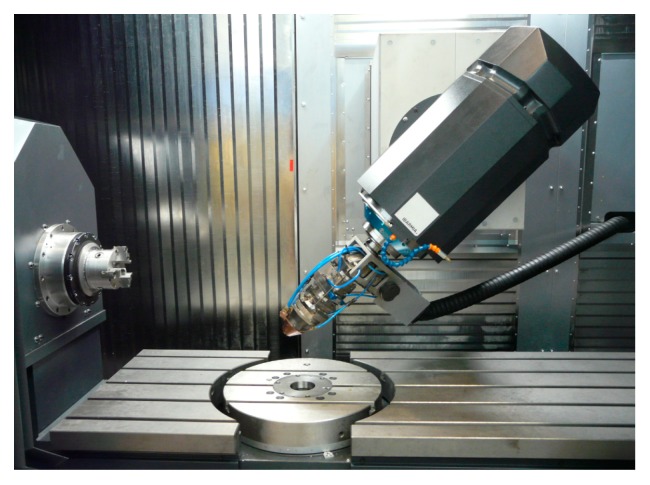
ZVH 45/1600 hybrid additive/subtractive hybrid machine.

All tests were performed in a ZVH 45/1600 hybrid additive/subtractive machine (Figure 1), from the manufacturer Ibarmia (Azkoitia, Spain). This multiprocess machine combines the LMD technology with 5-axis milling and turning capacity. It is also equipped with a Precitec YC52LMD coaxial head (Precitec GmbH & Co. KG, Gaggenau, Germany) with the possibility of assembling annular or 4-stream powder feed modules. The system also uses a Sulzer Metco TWIN-10-C powder feeder (Oerlikon Metco, Pfäffikon, Switzerland) and an Yb-Fiber Rofin FL030 laser generator (Coherent Inc., Santa Clara, CA, USA) of 3 kW with a continuous wavelength of 1.07 μm. The laser head features a focusing screw that offers the possibility of moving the collimating optics up to 10 mm in the focusing direction of the optics changing the laser beam diameter in the powder focus.

As previously mentioned, two different walls made of nickel-based alloy 718 were tested: one of them manufactured by an LMD process and another one produced by a conventional forging process. For the first wall, an M-328.95 powder supplied by FST (Flame Spray Technologies BV., Duiven, The Netherlands) was selected as a filler material. This powder is gas-atomized and has a particle size distribution between 45 and 150 µm. For the forged wall, a sol-annealed 718 alloy with a hardness of 89 HRB was selected, and it was also used as substrate for the LMD process. Table 1 shows the chemical composition of the material for the material in powder and forged state.

**Figure 2:**
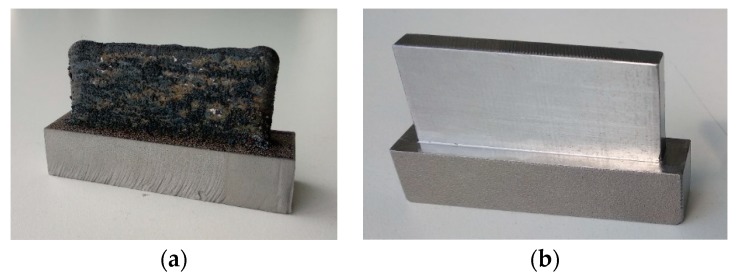
Walls manufactured by: (**a**) LMD-AD; (**b**) forged-SA.

The wall produced by LMD was manufactured using a power of 2500 W, a feed rate of 500 mm/min, and a powder mass flow rate of 20 g/min. It was composed of 30 layers and 2 beads, which were overlapped at 50%. After metal deposition, the workpiece width and height were measured by using a manual dial gauge inside the machining center, and the obtained values (22.2 mm height, 52 mm length, and 6 mm width) were considered as a reference in order to manufacture the forged wall. In Figure 2, the used workpieces are shown. Figure 2a displays the wall manufactured by LMD just after deposition (LMD-AD), and Figure 2b shows the sol-annealed forged (Forged-SA) wall after a milling operation.

### 2.2. Machining Strategy and Cutting Tools

Before the tests, both the LMD and the forged walls were pre-machined to obtain a final dimension of 49 × 18 × 3 mm^3^. The milling cutter used for this purpose (and also for the experimental tests) was a 4-flute endmill, with unequal helix angles and 10 mm in diameter, from the manufacturer KENDU (Segura, Spain). The characteristics of this endmill are shown in Table 2.

**Figure 3:**
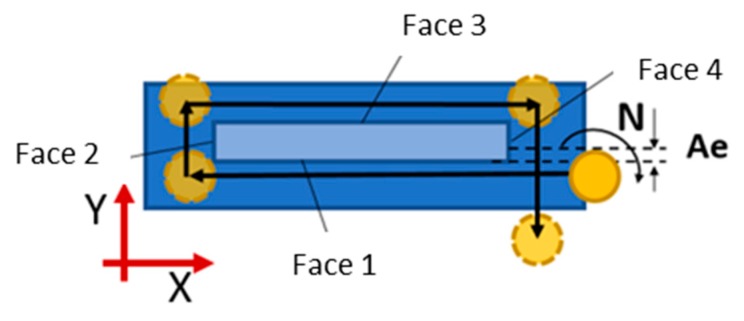
Tool path for the tangential milling.

In this experimental investigation, two sets of experiments were performed. Firstly, the tangential milling operation was studied using a radial depth of cut (A_e_) of 0.75 mm and an axial depth of cut (Ap) of 21.2 mm. The feed rate (F) and the spindle speed (N) remained constant at 300 mm/min and 2500 rpm, respectively, and external lubrication was used during the machining process. Figure 3 shows the tool path for this tangential milling operation.

**Figure 4:**
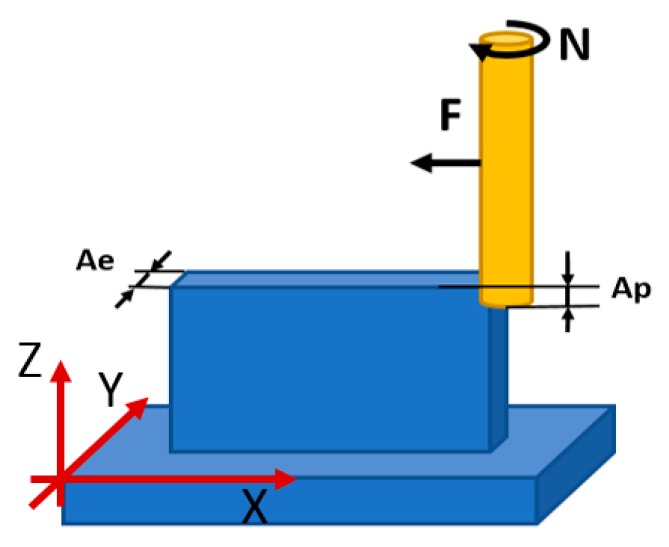
Tool path for the face milling.

In a second set of experiments, a face milling operation was performed in the upper surface of the workpiece (as shown in Figure 4). In this case, three passes were done using an axial depth of cut of 1.2 mm for the first one and 1 mm for the second and the third pass. The feed rate (F) and the spindle speed (N) also remained constant at 300 mm/min and 2500 rpm, and the tests were done with external lubrication and under flooded conditions. 

During the tests, cutting forces were registered using a Pro-micron Spike 1.2^®^ system (Pro-micron GmbH, Kaufbeuren, Germany). This is a sensorized tool-holder that can collect the bending moment in the X and Y directions, as well as the axial force and torque. It should be noted that during the milling operation, the direction of the cutting force on the tool holder is constantly changing. Thus, the bending moment of the tool holder, which equals the product of the cutting force and the moment arm, is more suitable for analysis compared with the cutting force and can directly reflect the current machining condition [21]. Thus, the bending moment signals in the X and Y directions were collected during the machining process.

The post-treatment analysis of the acquired signals was done by means of Python 2.7.6. ® software. Furthermore, the geometry of the walls was measured before and after the end-machining inside the hybrid machine. This allowed us to compare the final geometry of the walls with the initial one, and thus enabled the calculation of the removed material. In order to do so, a structured light technology camera from Texas Instruments (Dallas, TX, USA) and the LED projector uEye UI-3360CP (IDS, Obersulm, Germany) was used. The post-treatment of the data was done using DAVID 4 image analysis software. 

### 2.3. Workpiece Quality Analysis

**Figure 5:**
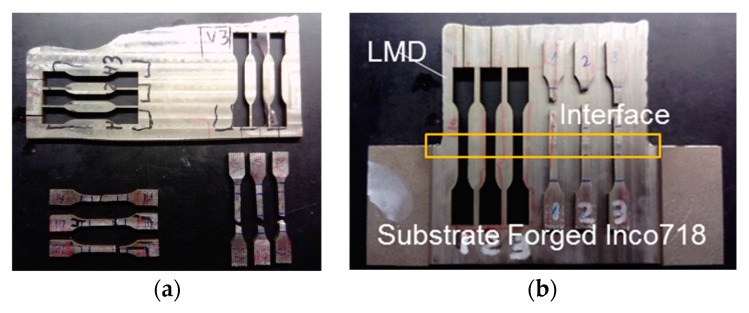
Specimens for tensile tests extracted from laser metal deposition (LMD) walls: (**a**) specimens in horizontal and vertical direction for evaluating the added material; (**b**) specimen for evaluating the union between the wall and the substrate material.

After the tests, the metallurgical and mechanical properties of the workpieces were studied. First, the walls were cut, ground, polished, and etched with an oxidic acid solution so as to measure the hardness and to analyze the metallic microstructure. This last analysis was done in the XY, XZ, and YZ planes (see Figure 4). Finally, with the aim of evaluating the mechanical properties of the wall obtained by LMD, a new wall of 180 × 120 × 6 mm^3^ was manufactured. From this workpiece, 6 tensile specimens with a section of 4 × 4.2 mm^2^ were extracted in different positions and directions, as shown in Figure 5a. In addition, for the evaluation of the union between the added and the substrate material, a wall of 120 × 70 × 6 mm^3^ dimensions was also manufactured using a 70-mm thick substrate, from which another 3 tensile specimens with a section of 5 × 4 mm^2^ were extracted, as indicated in Figure 5b.

## 3. Results

### 3.1. Mechanical Properties and Workpiece Microstructure Characterization

**Figure 6:**
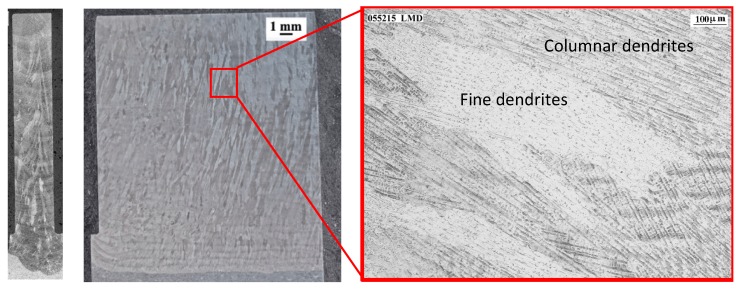
Macrographs and microstructure of the wall manufactured by LMD.

**Figure 7:**
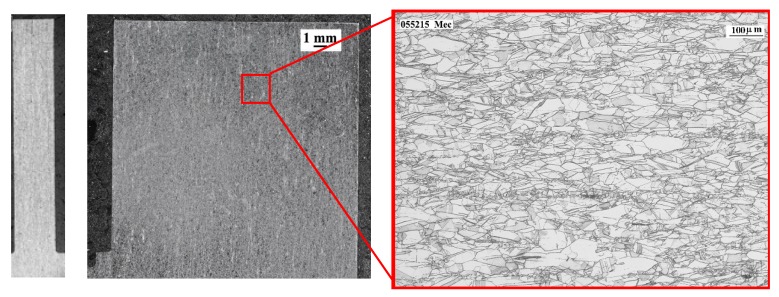
Macrographs and microstructure of the wall manufactured by forged-SA.

Figure 6 reveals the microstructure of the workpiece deposited by LMD. On the left side of the image, two macrographs of the part are displayed in the XZ and YZ planes of the wall, and on the right side, the microstructure of Inconel 718 is shown. Long columnar grains of several millimeters are observed in the micrographs, which are related to the heat input and cooling directions. The microstructure analysis also reveals the presence of a cellular dendrite structure with columnar dendrites and fine dendrites. In Figure 7, the microstructure of the wall produced by the forged-SA process is shown. In this case, the typical equiaxial grains with a grain size of ASTM-7 are observed. The elongation of the grains (produced by the induced plastic deformation in the forge process) is also noticeable.

**Figure 8:**
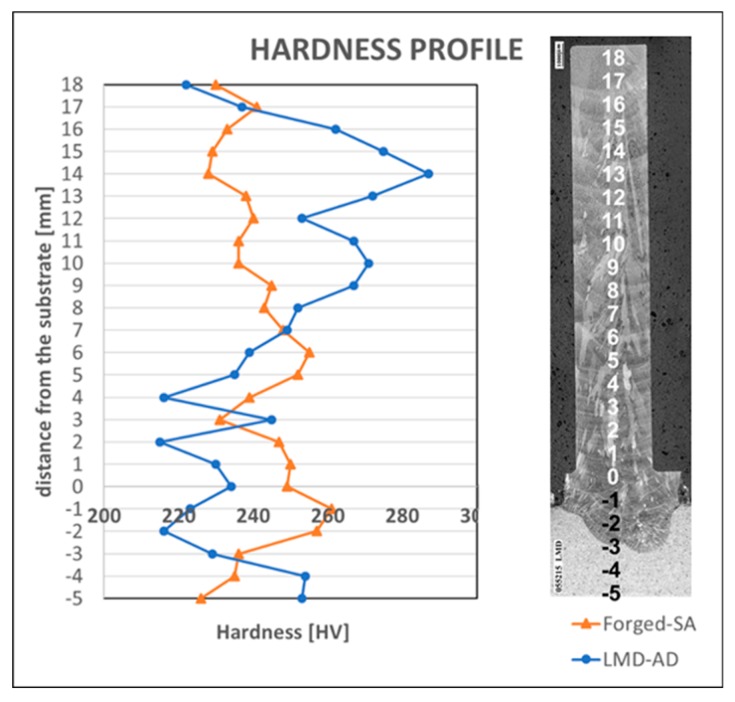
Hardness profiles of the walls.

Figure 8 shows the hardness profiles for both the forged wall and the one manufactured by LMD. The hardness indentations that correspond to the machined zone are those between 1 mm and 18 mm from the substrate. The results showed that the average value of the machined zone was of 231.6 HV in the case of forged-SA wall and of 251.8 HV in the case of the LMD-AD wall. Moreover, less variation of the hardness along the height of the wall was observed in the forged-SA workpiece. In this case, the highest deviation from the mean hardness value was under 7%, while in the LMD workpiece, it was of 14%. This occurs because in the manufacturing of each layer of the LMD wall, the laser heat input acts in a similar way to a heat treatment, thus affecting the material structure, grain growth, and hardness. For example, the smaller grain size observed in the LMD wall at a distance between 10 and 18 mm from the substrate could be responsible for the higher workpiece hardness in this area of the workpiece.

**Figure 9:**
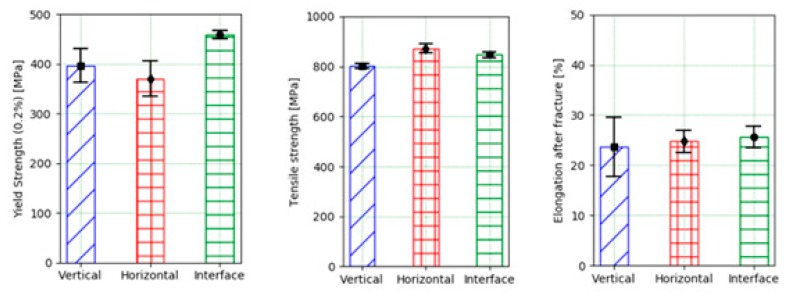
Mechanical properties of the LMD wall.

As was described in the previous section, tensile tests were also performed on the LMD wall in order to obtain the yield strength, the tensile strength, and the elongation at fracture. As shown in Figure 5, the mechanical properties were analyzed in three different directions: along the deposition path (named “horizontal direction” in Figure 5), in the vertical direction, and also in the interface between the substrate and the deposited material. As can be seen in Figure 9, the influence of the position of the specimen in the wall was very small. In fact, the highest variation observed for the studied mechanical properties was always under 10%.

On the other hand, the mechanical properties of the forged-SA material (according to the technical data provided by the manufacturer, (Huntington Alloys Corporation, Riverside Drive, WV, USA) are a yield strength (0.2%) of 430 MPa, a tensile strength of 869 MPa, and an elongation of 54.5%. The yield strength and tensile strength values were comparable to the ones provided by the workpiece produced by LMD. However, the elongation at fracture was much higher for the forged workpiece. This effect could be explained by the dendritic microstructure of the LMD workpiece. In addition, to analyze the composition of the material once the addition process by LMD was completed, optical emission spectrometry was carried out using a machine of the brand ARL, model 3460 (Thermo Fisher Scientific, Waltham, MA, USA). Table 3 shows the results obtained that show a greater amount of Mo.

### 3.2. Chip Morphology Analysis

**Figure 10:**
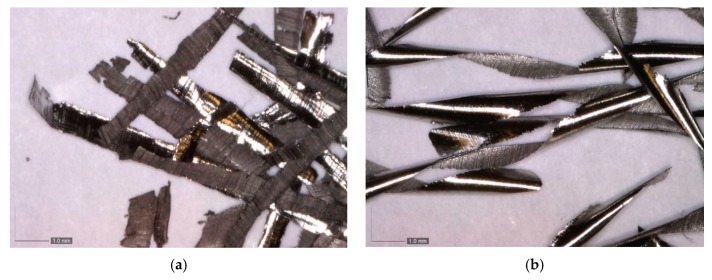
Chips obtained during end milling of walls: (**a**) LMD-AD; (**b**) forged-SA.

The chips obtained during the end milling of the different walls are shown in Figure 10. For both walls, the free surface showed a serrated and rough appearance that can be attributed to the shearing actions and large plastic deformations. However, the chips were significantly different in terms of length and shape. The chips generated in the machining of the LMD wall were shorter and straighter as compared to those obtained for the forged wall (which also had a spiral shape). This difference can be explained by the much higher value of elongation at fracture for the forged wall.

### 3.3. Cutting Forces

As mentioned, the face milling process was studied in the first set of experiments. It is important to consider that the thickness of the piece is not uniform after the LMD process, so the amount of material to be removed will be different in each area of the piece. As a consequence, the forces will vary considerably with the amount of material removed during the first milling pass. Under these circumstances, an optical measurement of the piece is of great help to know the initial dimensions of the wall and to determine the axial depth of cut to be used in the first pass of the machining process.

**Figure 11:**
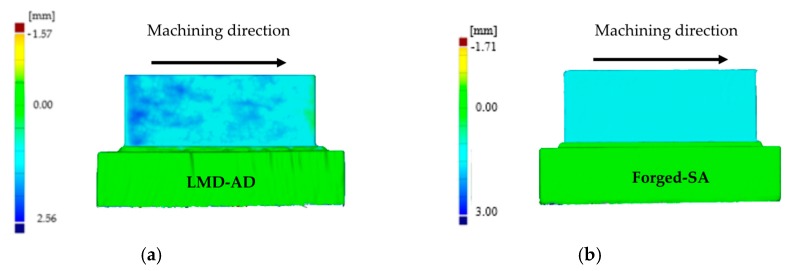
Radial depth of cut for the first set of experiments (face 1): (**a**) LMD-AD wall; (**b**) forged-SA wall.

**Figure 12:**
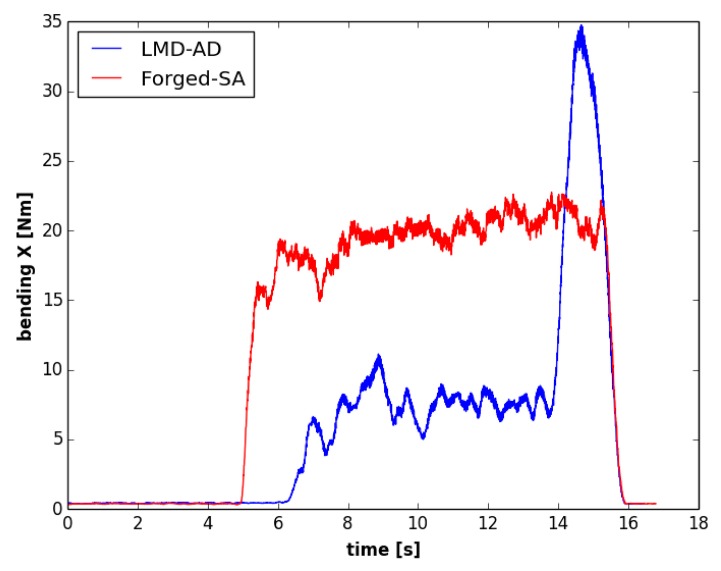
Bending moment evolution over time for face 1 in the first set of experiments (first pass).

In Figure 11a,b, the amount of material removed in the radial direction of the tool is shown for both the LMD and the forged walls, respectively. Figure 12 shows the evolution of the bending moment over the process time. For the wall manufactured by LMD, the depth of cut was nearly zero at the beginning of the machining process (green color in Figure 11a), and it increased gradually, reaching its maximum value of about 2.56 mm at the end of the milling pass (in dark blue in Figure 11a). The evolution of the bending moment was in accordance with the magnitude of the amount of material that has been removed. Regarding the forged-SA wall, the depth of cut was maintained nearly constant, as well as the bending moment signal. Similar trends were observed for the bending moment signal in the Y axis and for the torque.

**Figure 13:**
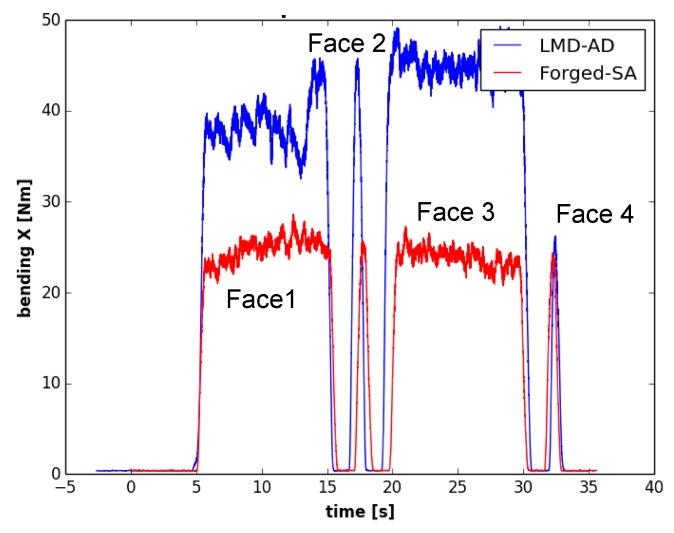
Bending moment evolution over time in the first set of experiments (second pass).

As can be seen in Figure 13, the bending moment was maintained approximately constant for both walls during the second face milling pass. In this operation, depths of cut were almost constant for the whole process. Nevertheless, the average bending moment observed for the second pass of the tangential milling operation was around 40% higher in the LMD-AD wall than in the forged-SA one. This effect can be related to the specific microstructure and mechanical properties of each material. Previous works have observed that a columnar microstructure with a higher grain size, such as the one observed for the LMD wall (Figure 7), leads to a lower machinability [18]. Furthermore, the higher cutting forces would also be related to the mean hardness value of the Inconel 718 in this wall, which was 10% higher as compared to the forged workpiece).

**Figure 14:**
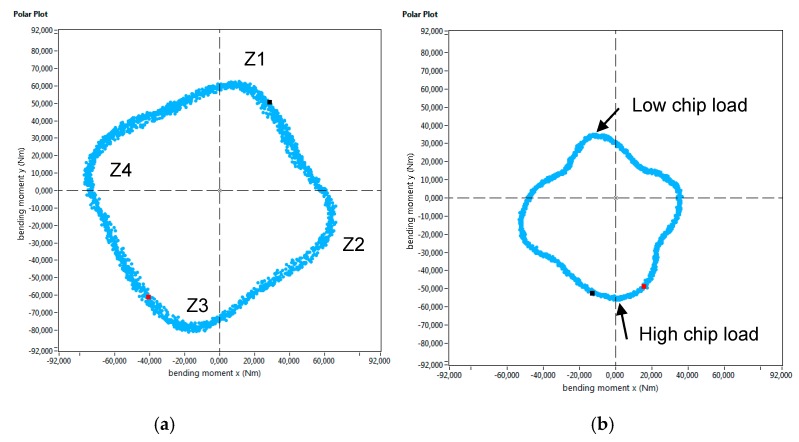
Polar plot of the average bending moment in the first set of experiments (second pass).

Furthermore, the Spike^®^ measuring system registers the bending moment both on the X and Y axis over time. If the intersection of these bending moments is displayed in a 2D graph (as shown in Figure 14), the chip load on a cutter at a given point of time can be determined. This graph is known as “polar plot”. If the mean value of the evolution of the bending moment over the whole machining process is depicted in such way, the condition of each cutting edge can be precisely defined. This tool can be used, for example, to determine a non-uniform wear of the milling cutter. Figure 14 shows the average polar plot of for the first set of experiments, and the four cutting edges are identified as Z1, Z2, Z3, and Z4. As has been commented before, the magnitude of the bending moments was higher for the wall produced by the LMD process, and it led to a much more open geometry. Moreover, from the polar plot in Figure 14b, it can also be concluded that the chip load on the 4 cutting edges of the mill was not uniform.

**Figure 15:**
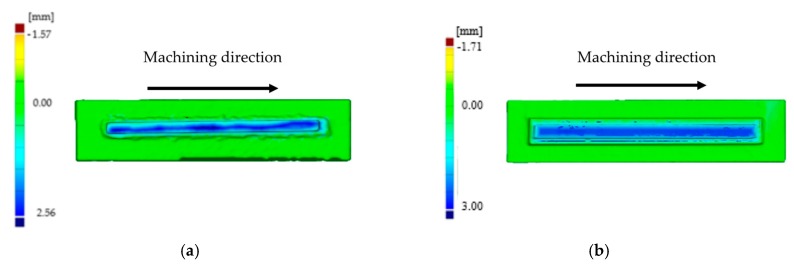
Axial depth of cut for the second set of experiments: (**a**) LMD-AD wall; (**b**) forged-SA wall.

**Figure 16:**
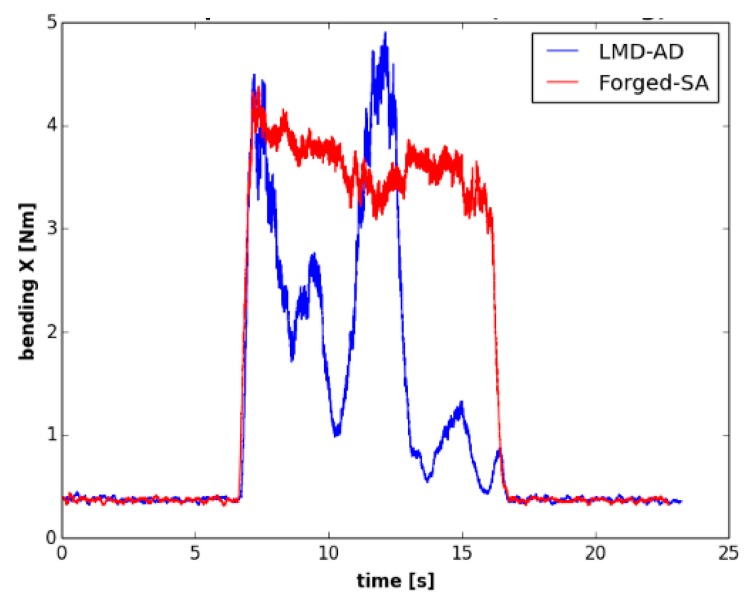
Bending moment evolution over time for the second set of experiments (first pass).

**Figure 17:**
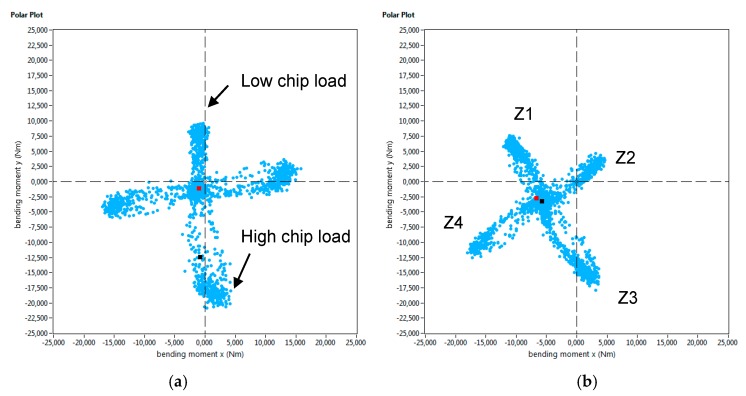
Polar plot of the average bending moment in the second set of experiments.

The evolution of cutting forces was also studied in the second set of experiments. Figure 15 shows the axial depth of cut measurements carried out with the optical system, and in Figure 16, the evolution of the bending moment is shown for both walls. Again, the evolution of the bending moment is in accordance with the magnitude of the depth of cut. For the LMD-AD workpiece (Figure 15a), the maximum bending moment and depth of cut values were obtained at the beginning and at the middle of the milling process. For the forged-SA wall (Figure 15b), both the axial depth of cut and the bending moment were constant. The polar plots for these tests are shown in Figure 17. As could be seen previously in the peripheral milling operation (Figure 14), the machining of the LMD wall showed the highest bending moments. The non-uniform chip load was also noticeable at the machining of the LMD wall. 

### 3.4. Influence of the Machining Process on the Final Workpiece Microstructure

**Figure 18:**
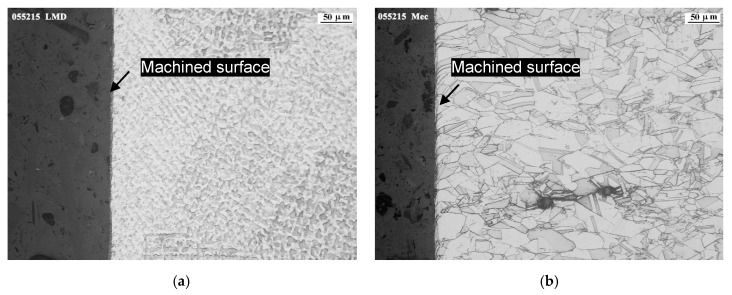
Cross-cut metallography images for: (**a**) milling LMD-AD, (**b**) milling forged-SA.

The analysis of the grain structure after tangential milling was carried out in the direction perpendicular to feed movement. As shown in Figure 18, a distorted surface layer on both materials was observed. The depth of the affected zone was not greater than 5 microns in case of LMD-AD milling and of about 15 microns in case of forged-SA. The machining effect on the surface was observed especially in case of milling forged-SA as it could be seen on the deformation in areas close to the surface. It is important to note the fading of grain boundaries produced in the machined affected layer, more visible in case of forged-SA due to its solution annealed microstructure in comparison with the dendritic structure of LMD-AD. This phenomenon was previously noted by Ahmed et al. [19] and was attributed to the high levels of deformation. It is deduced that the greater deformation observed in the case of the forged-SA is due to its lower surface hardness and greater ductility.

## 4. Conclusions

In this work, the influence of the LMD process on the end milling of Inconel 718 was studied and was been compared to the machining of a forged workpiece. Based on the obtained results, the following conclusions can be drawn:

1. No significant differences were observed in the mechanical properties provided by the specimens obtained at different positions of the wall manufactured by LMD. The yield strength and tensile strength values were comparable to the ones provided by the forged workpiece. However, the elongation at fracture was much lower for the LMD workpiece as compared to the forged one.
2. The chip geometry was significantly different in terms of length and shape. They were shorter and straighter for the LMD wall as compared to those obtained for the forged wall (which also had a spiral shape). This difference can be related to the higher elongation at fracture of the forged material.
3. The evolution of the bending moment is in accordance with the magnitude of the amount of the removed material. The higher values observed for the LMD can be related to its higher hardness value as compared to the forged material. Furthermore, the use of a bending moment polar plot has shown to be useful to determine a non-uniform loading of the milling cutter.

## Figures and Tables

**Table 1:** Chemical composition (wt.%) of the base and the powder material.

	Sol-Annealed Alloy 718	Powder Ni-Based 718
Fe	17.69	Bal
Co	0.22	0.33
Cr	18.67	18.99
Mo	2.88	3.15
Nb & Ta	5.0104	5.04
Ti	0.94	1.12
Ni	53.53	50.40
C	0.03	0.05
Mn	0.09	0.11
S	0.001	0.011
Si	0.08	0.10
Cu	0.12	0.02
Al	0.58	0.58
B	0.002	<0.006
P	0.010	0.01
Pb		<0.001
Se		<0.005
Others		<0.10

**Table 2:** Characteristics of the endmill.

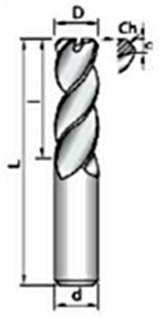	D [mm]	10
	l [mm]	22
	L [mm]	72
	Z [-]	4
	c [mm]	0.2
	Ch 45° [mm]	0.15

**Table 3:** Chemical composition of the powder material and the LMD-AD material.

	Powder Ni-Based 718		LMD-AD
Fe	Bal (~19.98)		17.1
Co	0.33		0.23
Cr	18.99		17.8
Mo	3.15		3.8
Nb & Ta	5.04		4.8
Ti	1.12		0.62
Ni	50.40		54.4
C	0.05		<0.02
Mn	0.11		0.30
S	0.011		<0.01
Si	0.10		0.49
Cu	0.02		0.05
Al	0.58		0.19
B	<0.006		0.005
P	0.01		0.015
Pb	<0.001		
Se	<0.005		
Others	<0.10		
